# The distribution of maternity services across rural and remote Australia: does it reflect population need?

**DOI:** 10.1186/s12913-017-2084-8

**Published:** 2017-02-23

**Authors:** Margaret I Rolfe, Deborah Anne Donoghue, Jo M Longman, Jennifer Pilcher, Sue Kildea, Sue Kruske, Jude Kornelsen, Stefan Grzybowski, Lesley Barclay, Geoffrey Gerard Morgan

**Affiliations:** 1 University Centre of Rural Health, University of Sydney, PO Box 3074, Lismore, NSW 2480 Australia; 2 Mater Research Institute, Women’s Health and Newborn Services, Mater Health Service, The University of Queensland (UQ), School of Nursing, Midwifery and Social Work, UQ, Brisbane, QLD 4101 Australia; 3 Institute for Urban Indigenous Health, School of Nursing, Midwifery and Social Work, UQ, Bowen Hills, QLD 4006 Australia; 4 Centre for Rural Health Research, Department of Family Practice, University of British Columbia, Vancouver, BC Canada

**Keywords:** Healthcare disparities, Rural health services, Geographic information systems, Catchment area (health), Maternity Hospitals, Health services research

## Abstract

**Background:**

Australia has a universal health care system and a comprehensive safety net. Despite this, outcomes for Australians living in rural and remote areas are worse than those living in cities. This study will examine the current state of equity of access to birthing services for women living in small communities in rural and remote Australia from a population perspective and investigates whether services are distributed according to need.

**Methods:**

Health facilities in Australia were identified and a service catchment was determined around each using a one-hour road travel time from that facility. Catchment exclusions: metropolitan areas, populations above 25,000 or below 1,000, and a non-birthing facility within the catchment of one with birthing. Catchments were attributed with population-based characteristics representing need: population size, births, demographic factors, socio-economic status, and a proxy for isolation - the time to the nearest facility providing a caesarean section (C-section). Facilities were dichotomised by service level – those providing birthing services (birthing) or not (no birthing). Birthing services were then divided by C-section provision (C-section vs no C-section birthing). Analysis used two-stage univariable and multivariable logistic regression.

**Results:**

There were 259 health facilities identified after exclusions. Comparing services with birthing to no birthing, a population is more likely to have a birthing service if they have more births, (adjusted Odds Ratio (aOR): 1.50 for every 10 births, 95% Confidence Interval (CI) [1.33-1.69]), and a service offering C-sections 1 to 2 h drive away (aOR: 28.7, 95% CI [5.59-148]). Comparing the birthing services categorised by C-section vs no C-section, the likelihood of a facility having a C-section was again positively associated with increasing catchment births and with travel time to another service offering C-sections. Both models demonstrated significant associations with jurisdiction but not socio-economic status.

**Conclusions:**

Our investigation of current birthing services in rural and remote Australia identified disparities in their distribution. Population factors relating to vulnerability and isolation did not increase the likelihood of a local birthing facility, and very remote communities were less likely to have any service. In addition, services are influenced by jurisdictions.

**Electronic supplementary material:**

The online version of this article (doi:10.1186/s12913-017-2084-8) contains supplementary material, which is available to authorized users.

## Background

Equitable health outcomes are defined by the World Health Organization as “*the absence of avoidable or remediable differences among groups of people, whether those groups are defined socially, economically, demographically, or geographically”* [1]. Achieving such equity in providing health services is an aspirational goal of the World Health Organization [2] and the Australian people [3, 4].

In Australia, 31% (*n* = 6,879,573) of the population live in rural and remote regions [5]. The population in these areas have a higher fertility rate and a higher perinatal mortality rate [6, 7]. Their life expectancy is up to 4 years lower than those in cities and prevalence of diabetes, high cholesterol, cancer or ischaemic heart disease also increases as remoteness increases [8]. An Australian review of health services in rural areas reported poor access to services, inferior quality of services compared to metropolitan services and fewer services in rural areas. It also reported that those scarcer services were unevenly distributed [9]. Australian research has highlighted the role of factors such as isolation, Indigeneity, and socioeconomic status (SES) in determining the location and service level of health facilities [10]. The most important influence on health in Australia is Indigeneity and this is true for all measures of health when compared to non-Indigenous Australians [11]. Aboriginal and Torres Strait Islander peoples make up 2.3% of the population^,^ but they have 4.5% of all births. This rises to 11.6% of births in rural and remote Australia [12] and 24% in remote or very remote locations [13].

Many factors influence the maternity service level [14] provided in rural and remote health facilities and there is debate about the appropriate level of service offered in some areas [15–17]. A consortium of the leading maternity care and rural organizations in Australia released a National Consensus Framework for Rural Maternity Services in 2010 that is built on principles which include “to ensure that rural maternity services are equitable in terms of distribution and access” [18]. However, this in not reflected in outcomes [19].

The Australian government funds health services via agreements with its eight jurisdictions. A nation-wide plan, the Australian National Maternity Services Plan was also introduced in 2010 [20]. This plan acknowledged the poorer health outcomes for people living in rural and remote Australia, in particular Aboriginal and Torres Strait Islanders, and proposed action to address specific issues including to: “*examine tools … and develop a rigorous methodology to assist in future planning for maternity care, including in rural and remote communities"* [19].

A review of indices of rural health care found that access to services is influenced by such factors as the geographical placement of the facility, the isolation of the community and the socioeconomic vulnerability of the catchment population [10, 21, 22]. The Rural Birthing Index (RBI) is one such tool examining maternity services [23]. It was developed in British Columbia, Canada using data from focus groups and interviews to identify population derived indicators of maternity service need. Three were identified: the number of births to women living in the area; the vulnerability of the community measured by SES; and the isolation of the service as measured by the time taken to travel to the nearest facility that can perform a C-section at any time [24]. The current study is part of a larger project to assess the applicability of the RBI to the Australian context, and to systematically assess a measure of catchment need across rural and remote Australia.

This paper systematically estimates measures of the need of the population within the catchment for a facility across rural and remote Australia. Equitable distribution of services should include adjustments in the provision and level of services in response to population characteristics. The authors propose that increasing levels of service will be associated with increasing numbers of births (need); and other dimensions of need which were increasing disadvantage (SES) (vulnerability); increasing proportion of Aboriginal and Torres Strait Islanders in the population (vulnerability), and decreasing proximity to another facility capable of undertaking an emergency operative birth (C-section) (isolation). To do this, the association between existing birthing services and the characteristics of its populations will be modelled using geographically defined service catchments. This is an ecological study which aims to examine the association between population-based characteristics of need including vulnerability and isolation and the provision of maternity services across rural and remote Australia.

## Methods

To undertake this study, a geographical catchment was constructed around each health facility in Australia, and then population characteristics were assigned to that catchment to estimate population based indicators of maternity service need. This enabled two stages of modelling to be undertaken.

### Catchments and travel times

A health facility catchment is the surrounding geographical area bounded by a one-hour, road-based travel time using a vector-based network analysis approach within a Geographic Information System (GIS) environment similar to Schuuman [24] and provides the basis for determining population level characteristics. Australian road network maps from Geoscience Australia GEODATA TOPO 250 K Series 3 (packaged in ARCMAP format) [25] were used to assign road speeds as a function of road type and surface [26] as defined in the Australian Cardiac Aria Study. The Northern Territory (NT) has higher speed limits than other Australian jurisdictions, for example, Australian highways have a road speed of 100 km except in the NT where it is 135 km/h. Hence all the NT road speeds were increased by 25% to better reflect actual speeds. Defining catchments based on road travel produced irregularly shaped areas. To avoid double counting, catchment populations in adjacent health facilities that abut, catchments were split to create an equal travel time between the adjacent health facilities. Any non-birthing facilities within one-hour road travel time of an adjacent birthing facility was excluded to avoid inappropriately reducing the size of the catchment population for existing birthing facilities. All spatial data processing was conducted using ESRI ArcGIS 10.0 and 10.1 (ESRI ArcGIS 10.0) [27].

### Data overlays of population characteristics

The 2006 Australian Bureau of Statistics (ABS) census provides a range of socio-demographic data at nested spatial units including Statistical Local Areas (SLAs; *n* = 1,426), Census Collector Districts (CCDs, *n* = 38,704, about 220 households in cities which decreases with increasing rurality) [28] and MeshBlocks (MB = 347,000, around 30 to 60 households) [28]. SLA is the most common spatial unit used for routinely reporting detailed population data and birth registration data for all years, while more limited population and socio-demographic data is routinely available at the CCD or MB level for Census years (every five years).

To determine the number of people living in the catchment area, and the number of babies born to mothers in the area, routinely available data were accessed for birth registrations and for Estimated Residential Populations (ERP). Five year averages were used for 2005 to 2010 for each SLA throughout Australia [12]. Usual Residential Population (URP) for the 2006 census was available at MB level [29]. URP is considered a less precise estimate of residential population than ERP but is routinely available on a smaller spatial scale. MB data also includes information on non-residential land use within SLAs [12]. We weighted the SLA level birth numbers and ERP by mesh block URP in order to better estimate irregularly shaped catchment ERP and birth numbers while adjusting for non-residential land use [12]. The number and proportion of women of childbearing age (15 to 44 years) was estimated from URP data, and weighted by the proportion (area) of each CCD contained in the catchment area.

Socioeconomic status (SES) was measured by the ABS 2006 Socio-Economic Indexes for Areas Index of Relative Socio Economic Disadvantage (IRSD) [30]. The IRSD is based on 14 Census questions including household income, housing, Aboriginality and education, and is constructed using the CCD [28]. Low scores (decile 1) indicate the most disadvantage relative to the all Australia, and the highest score indicates least disadvantage (decile 10). To assign a SES score for each catchment the IRSD score (mean = 1,000, standard deviation = 100) was aggregated to each catchment and then converted to deciles.

The Aboriginal and Torres Strait Islander population was also estimated using URP data, weighted by the population proportion of each CCD in the catchment area. For the modelling, data was categorised into multiples of the national rate of Aboriginal and Torres Strait Islander persons (2.5%).

Degree of isolation of a facility was defined using road travel time in minutes to the next nearest facility that had the capability and staff to perform emergency caesarean section (C-section facility) [23] For modelling, it was categorised into intervals of 1 h (Stage 1) and half hour (Stage 2).

Rurality was defined using the ABS Remoteness Area Structure (RA) [28]. In this study, we refer to RA 1 as Major Cities; RA 2 (inner regional) and 3 (outer regional) as ‘rural’; and RA 4 (remote) and 5 (very remote) as ‘remote’.

### Health facility locations and maternity service levels

A health facility may or may not provide birthing services, influenced by a range of factors such as proximity to other birthing services, demand, or staff resource issues. Health facilities in rural and remote Australia provide a range of maternity services with some rural populations having local access to only hospital or community based antenatal and postnatal care. The location of health facilities in Australia was established using their geocoded location [latitude and longitude] sourced from the ‘My Hospitals’ web site [31] or the airport for seven very remote facilities. After catchments were defined for all facilities, exclusions were made if in RA 1 ‘major cities’, or in large regional centres (catchment population of more than 25,000) or the sparsely populated catchments of less than 1,000. Facilities offering birthing services were identified using jurisdictional lists of hospitals and perinatal reports for 2005-2010, and confirmed using the My Hospitals’ website [31, 32]. The level of maternity service for each health facility was provided or confirmed by representatives from the Maternity Services Inter-Jurisdictional Committee and are defined using the Australian National Maternity Service Capability Framework [14]. For this study, a health facility that does not provide birthing but may provide antenatal and/or post-natal care is a ‘non-birthing facility’ (Level 1, Table 1) [14]. A summary of the six levels of maternity service is provided in Table 1. The next level provides a birthing service, which requires dedicated equipment and staff to care for the mother and newborn (Level 2, a ‘birthing facility’). Birthing facilities were further divided by their capacity to perform an emergency caesarean section (C-section), an operative birth in specialist theatre that occurs after labour has commenced (Levels 3-6). Facilities that cannot provide this service are defined here as ‘non-C-section birthing’ (Level 2). Women with a high-risk pregnancy due to say, medical illnesses, need specialist care (Level 5, 6).

**Table 1:** National maternity service capability framework level of maternity service descriptors and definitions for modelling in both Stage 1 and 2 models [14]

Level of maternity service	Service descriptions	Stage 1 model birthing vs non-birthing		Stage 2 model non C-section birthing vs C-section	
No level or 1	No local birthing services, May have antenatal and postnatal care		non-birthing		
2	Local birthing services without C-section, Low risk births beyond 38 weeks’ gestation	birthing			Non C-section birthing
3	Local birthing services with C-section, Moderate risk births beyond 36 weeks’	birthing		C-section	
4	Local birthing services with C-section, Birthing beyond 34 weeks, Special Care Nursery	birthing		C-section	
Levels 5 and 6	Local birthing services with C-section, High-risk births, specialist services for mother and baby	birthing		C-section	

The six states of Australia, New South Wales (NSW), Victoria (Vic), Queensland (Qld), South Australia (SA), Western Australia (WA) and Tasmania (TAS) plus the NT are included as covariates in this study. The Australian Capital Territory was excluded as it is classified as metropolitan (RA 1). The catchment population range of 1,000 to 25,000 excluded all C-section facilities in TAS and left just two in the NT. When small numbers of facilities precluded analysis, the data for TAS and NT was combined with WA, which had a similar (50%) rate of birthing and non-birthing facilities - referred to as WAplus.

### Statistical modelling

A two stage binary logistic regression modelling approach was used to assess catchment level socio-demographic and service delivery factors associated with maternity service level for health facilities in Australia. Stage 1 investigated factors related to whether a health facility has a birthing service or not (see Table 1, non-birthing vs birthing). Stage 2 assessed the subset of facilities with a birthing service (Table 1, no C-section birthing vs C-section). Potential explanatory factors were categorised to ensure reasonable distributions for statistical modelling, which could result in different categorisations for each stage. The number of births were used both as a continuous measure (births divided by 10) for ease of interpretation, and in categories based on 50 birth increments. Model fit was assessed using odds ratios (OR) with 95% confidence intervals (95% CI) to measure effect size; the chi squared test (chisq) for the overall model fit and the Wald statistic for significance of each parameter; the model Nagelkerke pseudo R^2^ (denoted as R_Nag_^2^) reflected model improvement. Model discrimination was also assessed by percentage agreement, Area under the Curve (AUC), and the number of discordant facilities for each Stage. The importance of the covariates in each logistic model and the various parameterisation of the covariates were assessed based on the improvement to model fit, discrimination and the interpretability of the covariates. Results are reported as adjusted OR (aOR) and their corresponding 95% CI.

## Results

### Facilities

**Fig. 1:**
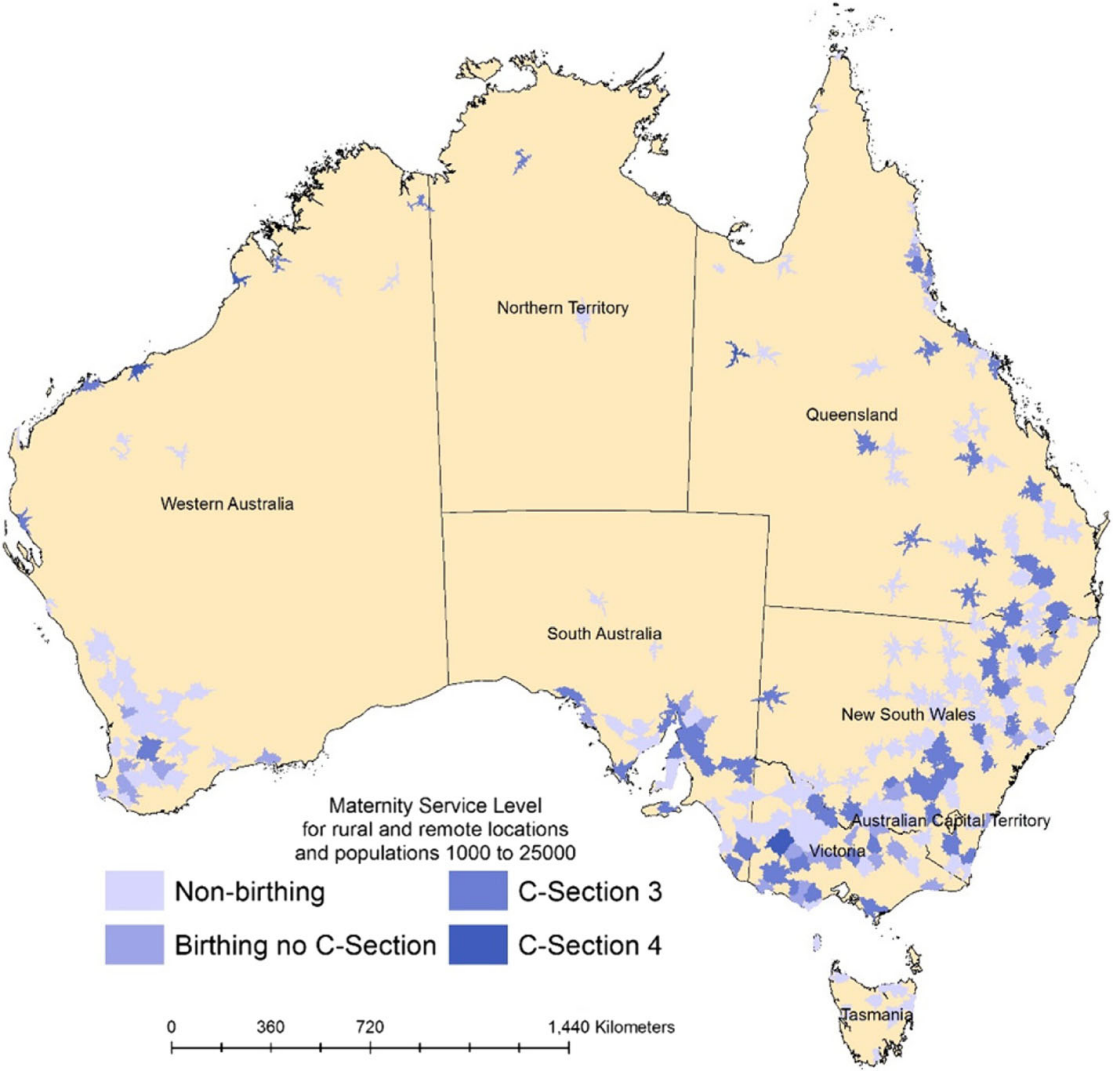
Health facilities in rural and remote Australia by level of maternity service

There were 259 health facilities identified in communities with one-hour catchment populations of 1,000 to 25,000 in rural and remote Australia. No health facility providing birthing services had a catchment of less than 1,000 people. The upper population limit of 25,000 excluded 58 rurally located, referral hospitals providing specialised services (levels 4-6). Birthing services were provided by 108 (42%) health facilities, and 73 (68%) of these had the capability to perform a C-section. Figure 1 presents the geographical distribution of the health facilities included in this study.

**Fig. 2:**
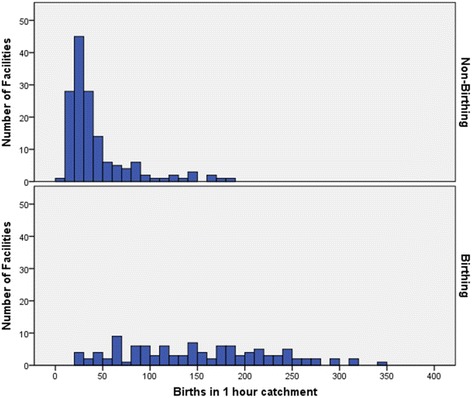
Annual birth numbers (5 year average) in catchments for non-birthing and birthing facilities

The histograms in Fig. 2 summarise the annual births (averaged for five years) in health facility catchments categorised by birthing or non-birthing. The range of births was from one to 350, but nearly half of the catchments had fewer than 50 women giving birth per year. There was a substantial overlap in the number of births in the catchments of non-birthing facilities and birthing facilities. In fact, eight percent of non-birthing facilities have more than 100 births per year and 10% of birthing facilities have less than 50 births per year. However, increasing birth numbers do have a significant association with the presence of birthing facilities (chisq = 132, df = 4, *p* < 0.001).

**Fig. 3:**
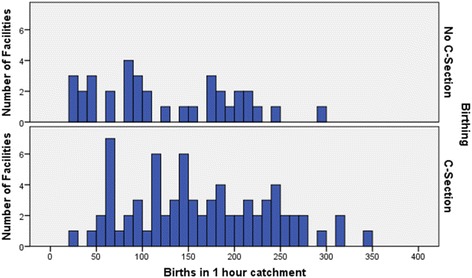
Annual birth numbers (5 year average) in catchments for no C-section birthing and for C-section facilities

Increasing catchment birth numbers (in categories of 50 births), have a significant association with a higher proportion of facilities offering C-section (chisq = 17.7, df = 4, *p* = 0.003). Non C-section birthing facilities exhibit a bimodal pattern with similar proportions below and above facilities with 100 births, whereas only 23% of C-section facilities have 100 births or less (Fig. 3).

**Fig. 4:**
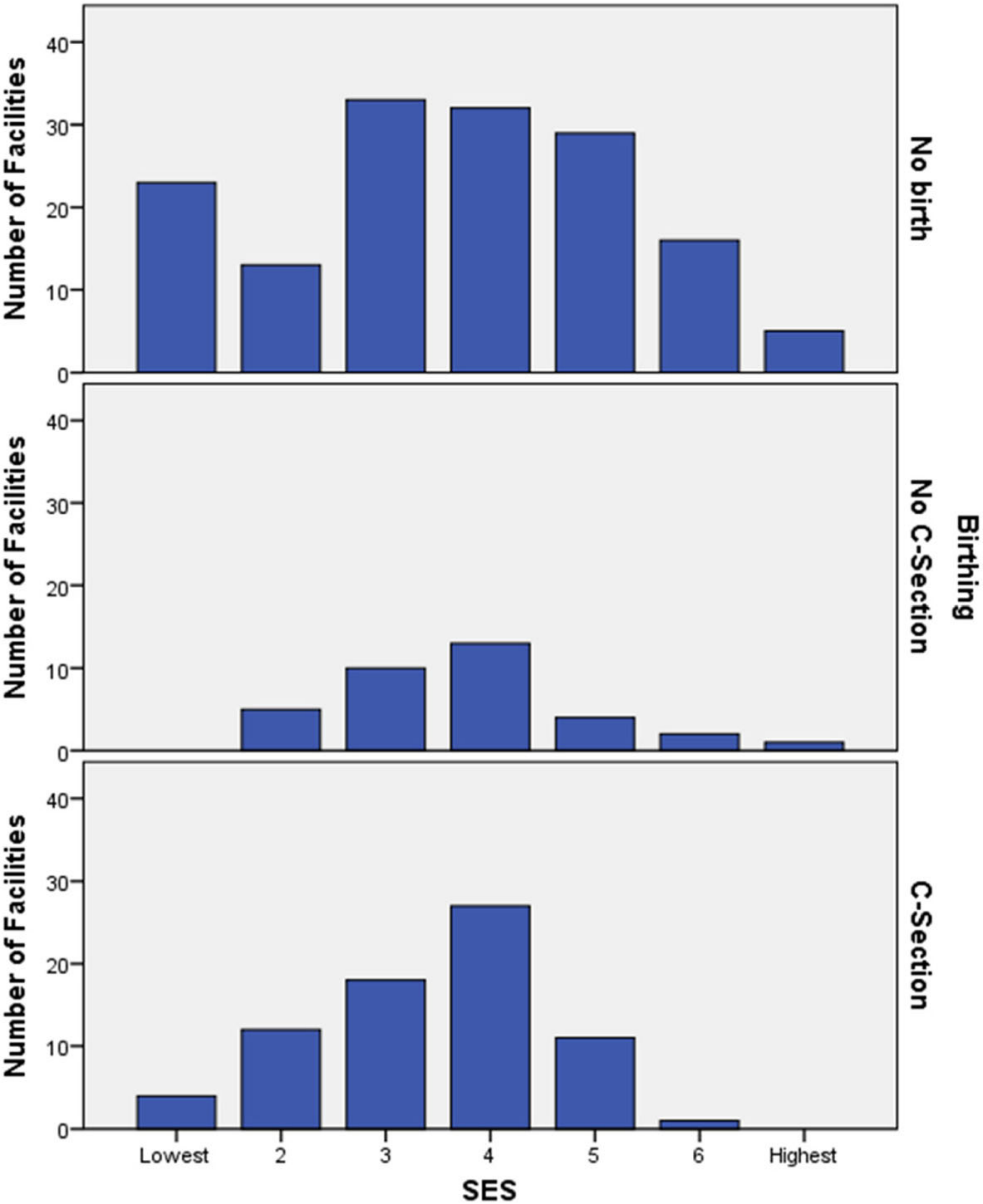
Numbers of facilities for No birthing, no C-section birthing and for C-section birthing by SES decile

The overall low SES in rural and remote Australia was represented in our study by the catchment IRSD decile scores. The health facilities in our study ranged from 1 (lowest or most disadvantaged), to just 7 (highest or least disadvantaged), (Fig. 4). Non-birthing facilities are evenly distributed over all deciles, but C-section birthing facilities were concentrated in the low-mid range (2-5; chisq = 27.3, df = 12, *p* = 0.007), (Fig. 4).

**Fig. 5:**
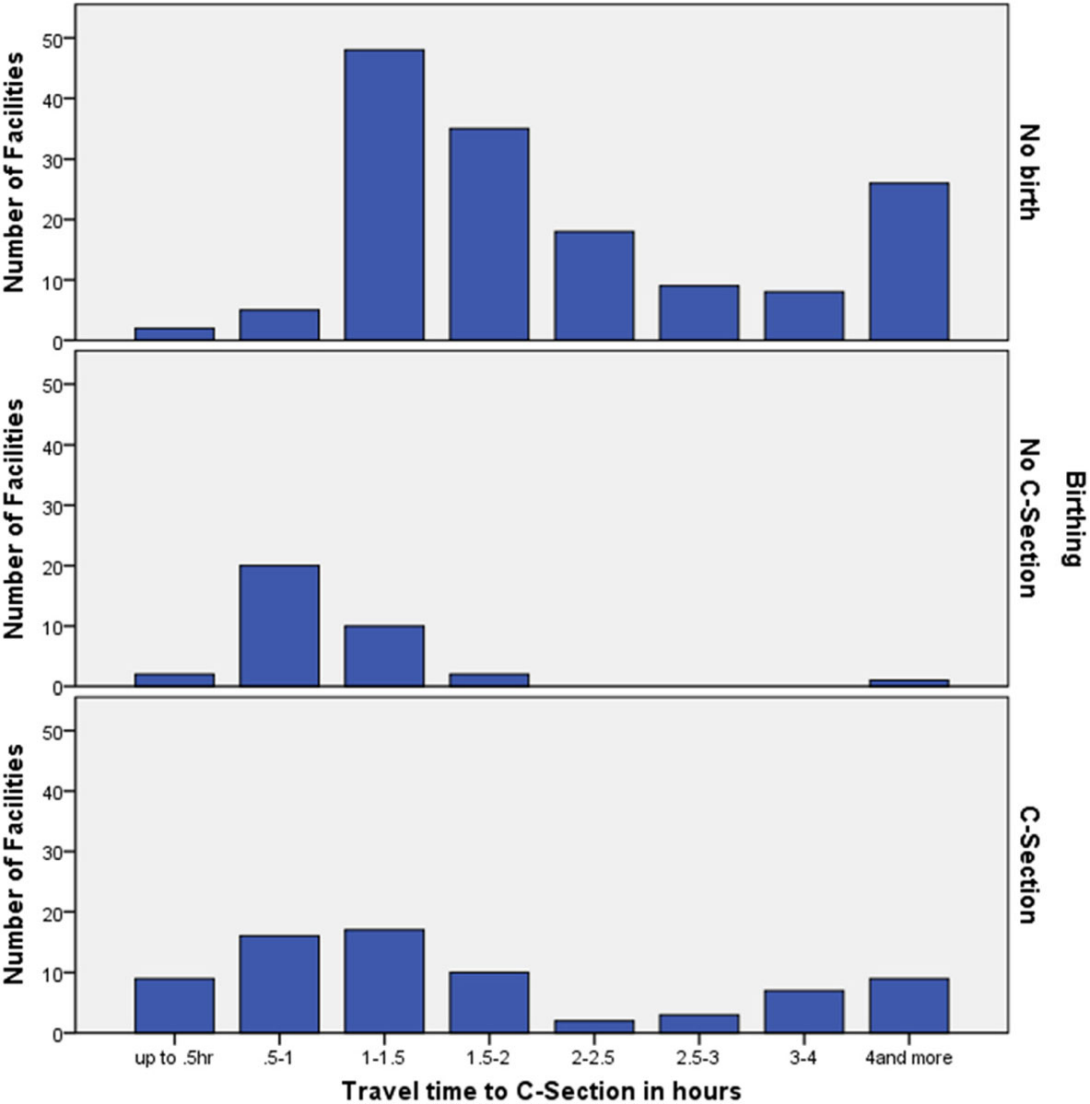
Numbers of facilities for No birthing, no C-section birthing and for C-section birthing by time to nearest C-section service

Over half (55%) of non-birthing facilities were 1 to 2 h from a C-section facility. Of the birthing facilities, 44% were within one-hour of a C-section and 80% were within 2 h of a C-section service. Almost all non C-section birthing facilities (97%) were within 2 h of a C-section facility as illustrated in Fig. 5.

**Fig. 6:**
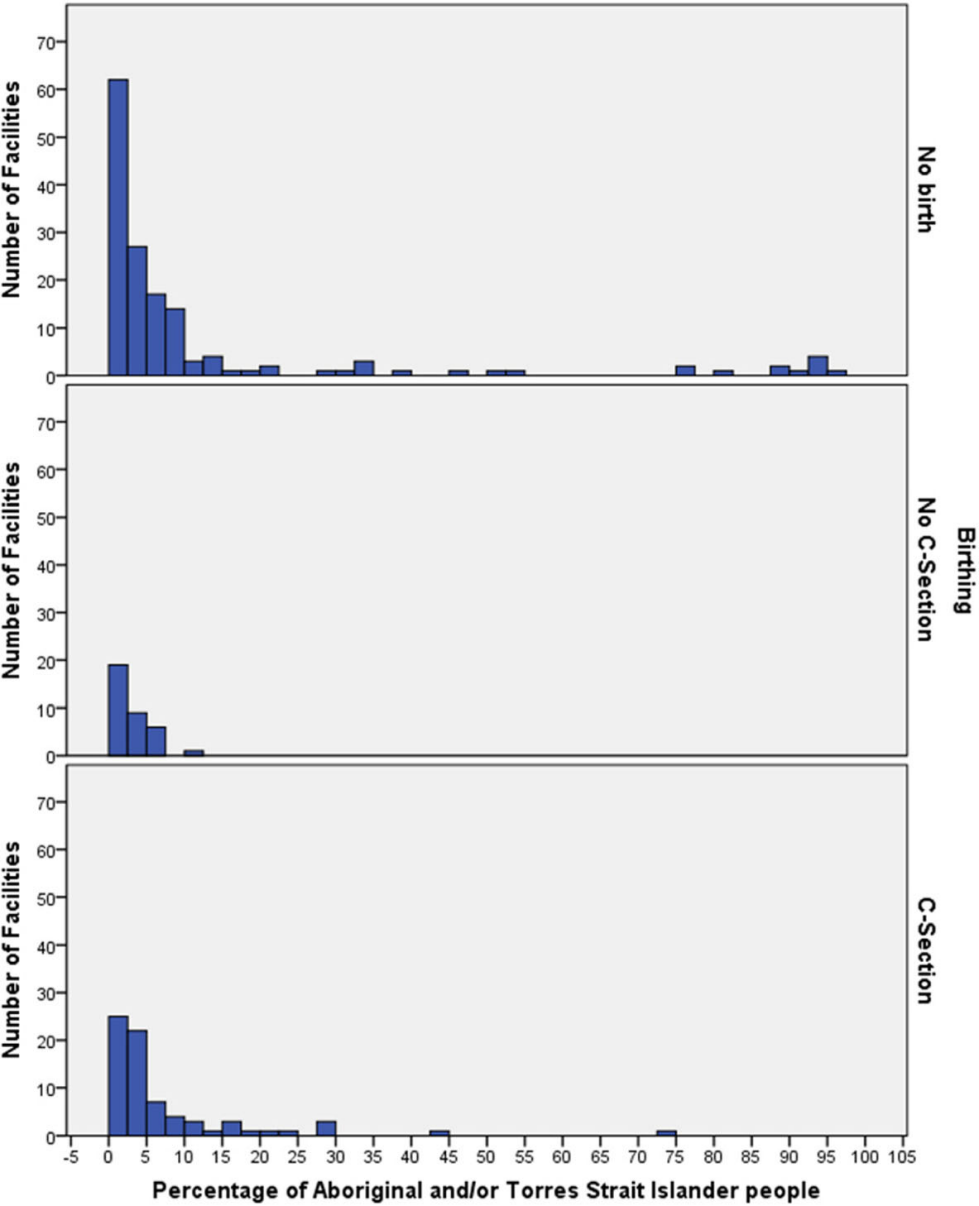
Numbers of facilities for no birthing, no C-section birthing and for C-section birthing by percentage of Aboriginal and Torres Strait Islander people in the catchment

There were 47 (18%) health facility catchments where more than 10% of the people were Aboriginal and Torres Strait Islander. Sixteen provided birthing services (15% of all birthing services), mostly (94%) providing a C-section. However, catchments with more than 10% of Aboriginal and Torres Strait Islander populations were more likely to (21%) be in non-birthing catchments, Table 2 and Fig. 6.

**Table 2:** Characteristics of facilities with catchment populations of 1,000 to 25,000, by service delivery level, 2005 to 2010

Catchment characteristics	Non-birthing		All birthing		Non C-section birthing		C-section birthing		Total	
	*n* = 151		*n* = 108		*n* = 35		*n* = 73		*n* = 259	
	Freq	(%)	Freq	(%)	Freq	(%)	Freq	(%)	Freq	(%)
Births (per year)										
< 50	116	(77)	11	(10)	9	(26)	2	(3)	127	(49)
50-100	23	(15)	23	(21)	8	(23)	15	(21)	46	(18)
100-150	8	(5)	22	(20)	4	(11)	18	(25)	30	(12)
150-200	4	(3)	8	(7)	7	(20)	1	(1)	25	(10)
> 200	0	(0)	9	(8)	7	(20)	2	(3)	25	(10)
SES (IRSD deciles)										
1 most disadvantaged	23	(15)	4	(4)	0	(0)	4	(5)	27	(10)
2	13	(9)	17	(16)	5	(14)	12	(16)	30	(12)
3	33	(22)	28	(26)	10	(29)	18	(25)	61	(24)
4	32	(21)	40	(37)	13	(37)	27	(37)	72	(28)
5	29	(19)	15	(14)	4	(11)	11	(15)	44	(17)
6, 7 least disadvantaged	21	(14)	4	(4)	3	(9)	1	(1)	25	(10)
Travel time										
≤ 1 h	7	(5)	47	(44)	22	(63)	25	(34)	54	(21)
1-2 h	83	(55)	39	(36)	12	(34)	27	(37)	122	(47)
2-3 h	27	(18)	5	(5)	0	(0)	5	(7)	32	(12)
3-4 h	8	(5)	7	(6)	0	(0)	7	(10)	15	(6)
> 4 h	26	(17)	10	(9)	1	(3)	9	(12)	36	(14)
Aboriginal & Torres Strait Islander										
< 2.5% national rate	62	(41)	44	(41)	19	(54)	25	(34)	106	(41)
2.5-5%	27	(18)	31	(29)	9	(26)	22	(30)	58	(22)
5-10%	31	(21)	17	(16)	6	(17)	11	(15)	48	(19)
10-25%	11	(7)	11	(10)	1	(3)	10	(14)	22	(8)
> 25% (10 x rate)	20	(13)	5	(5)	0	(0)	5	(7)	25	(10)
Remoteness										
Rural RA 2	14	(9)	36	(33)	15	(43)	21	(29)	50	(19)
Rural RA 3	73	(48)	55	(51)	18	(51)	37	(51)	128	(49)
Remote RA 4	39	(26)	12	(11)	2	(6)	10	(14)	51	(20)
Remote RA 5	25	(17)	5	(5)	0	(0)	5	(7)	30	(12)
Women aged 15-44										
mean (SD)	25.3	(5.8)	20.6	(2.6)	20.9	(3.0)	20.5	(2.4)	23.3	(5.3)
median	24.5		20.4		20.8		20.3		22.2

### Stage 1 Modelling - birthing facilities vs non-birthing facilities

For Stage 1, descriptive statistics and the tests of association are presented in Additional file 1: Table S1. The results of the univariable logistic models are presented in Additional file 2: Table S2. Results are presented as OR and their 95% CI and model fit and predictive accuracy parameters for the explanatory variables.

After assessing a range of models with increasing numbers of predictor variables, the “best” multivariable model for Stage 1, is presented in Table 3. The explanatory parameters are listed in order of the strength of their contribution to the model. Birth numbers (divided by 10) as a continuous input provided a better fit than categories based on 50 births. This model had 92% agreement between predicted birthing and non-birthing facility, a large AUC (0.97) and a high R_Neg_^2^ (0.79) with only 23 (out of possible 259) discordant sites. SES was retained based on an a priori decision to include this potentially important risk factor.

**Table 3:** Multivariable logistic Stage 1 model for catchments of birthing versus non-birthing facilities

Parameters	Wald	df	*p*	Adjusted OR	aOR 95% CI^a^
Birth numbers (divided by 10)	45.1	1	**0.000**	1.50	[1.33-1.69]
Travel time to C-section	16.4	4	**0.003**		
< 1 h (ref)				1.00	
1-2 h	16.2	1	**0.000**	28.7	[5.59-148]
2-3 h	0.19	1	0.664	1.44	[0.28-7.49]
3-4 h	0.45	1	0.504	2.19	[0.22-21.7]
4+ hours	0.68	1	0.408	2.15	[0.35-13.1]
SES (IRSD deciles)	6.44	3	0.092		
1 most disadvantaged	4.50	1	**0.034**	0.07	[0.01-0.82]
2-4 (ref)				1.00	
5	0.09	1	0.760	0.80	[0.18-3.45]
6-7 less disadvantaged	1.39	1	0.238	0.19	[0.01-3.00]
Jurisdiction	23.98	6	**0.001**		
NSW (ref)				1.00	
QLD	0.01	1	0.919	0.92	[0.20-4.20]
VIC	11.4	1	**0.001**	26.9	[3.98-182]
SA	10.7	1	**0.001**	20.5	[3.37-124]
WA	1.34	1	0.246	2.81	[0.49-16.1]
NT	0.36	1	0.550	0.36	[0.01-10.4]
TAS	5.30	1	**0.021**	0.03	[0.00-0.59]
Aboriginal & Torres Strait Islander	12.6	4	**0.014**		
< 2.5% (ref)				1.00	
2.5-5%	9.70	1	**0.002**	14.1	[2.67-74. 9]
5-10%	0.66	1	0.415	2.03	[0.37-11.1]
10-25%	5.99	1	**0.014**	15.9	[1.74-146]
> 25%	3.69	1	0.055	21.2	[0.94-479]

Overall Model fit Chisq = 231.0 df = 18 *p* < 0.001

^a^aOR 95% CI = Adjusted Odds Ratio 95% Confidence Interval

Bold indicates significant effects or significant aOR (*p* < 0.05)

The number of births to women living in the catchment was the strongest predictor for a facility having a birthing service, with the likelihood of a facility offering birthing increasing by 50% for every 10 births. Travel time to C-section was also associated with having a birthing facility. The large OR for between 1 to 2 h is an artefact of our exclusion of all non-birthing facilities within the one-hour catchment of a birthing facility.

### Stage 2: facilities offering birthing with or without C-section

For the stage 2 model the outcome measure was for all birthing facilities dichotomised as C-section facilities capability (levels 3-4; *n* = 73), compared to no C-section birthing facilities (level 2; *n* = 35). Descriptive characteristics of the facilities as well as results of tests of association and trend for Stage 2 are presented in Additional file 3: Table S3. Additional file 4: Table S4 provides the summarised results of univariable logistic regression and results are presented as OR and their 95% CI and model fit and predictive accuracy parameters for the explanatory variables.

Jurisdiction was significantly associated with a facility providing C-section compared not in the univariable analysis. Using NSW as the reference, Vic (OR = 0.31, 95% CI 0.10-0.98) was less likely to have C-section facilities whereas the likelihood of C-section facilities in QLD, SA and WAplus was not different to NSW, so a combination of jurisdictions were used in the multivariable logistic modelling process. SES was not significantly associated with the likelihood of a C-section facility. However, the proportion of Aboriginal and Torres Strait Islander persons was significant in both the continuous and categorical forms with a 15% increase in the likelihood of a C-section facility with every 1% increase in the Aboriginal and Torres Strait Islander population in the catchment.

The multivariate logistic model which maximized R_Neg_^2^ (0.59), AUC (0.91), and percentage agreement (79.6%) and minimised discordant sites (*n* = 22) is presented in Table 4. Various categorisation of the covariates were assessed and those shown in Table 4 provided a balance between the best model fit and interpretability of the covariates. All categories of birth numbers were 18 to 90 times more likely to have a C-section facility compared to a catchment of less than 50 births. Time to nearest C-section facility also retained significance. Where facilities with C-section were more than one to 1.5 h away from an alternative C-section, they were 4-5 times more likely to offer C-section compared to closer facilities. This increased to 15 to 80 times more likely to be C-section facilities for longer travel time. Jurisdictions in ‘NSW, QLD, SA’ were 11 times more likely to have a C-section facility in rural and remote areas compared to ‘VIC, WAplus‘. Although not significant in the final model, SES was retained based on an a priori decision to include this influential proxy measure for vulnerability, and did marginally improve the R_Neg_^2^, AUC and the number of discordant facilities.

**Table 4:** Multivariable logistic Stage 2 model for catchments of no C-section birthing facilities and C-section facilities

Parameters	Wald p	Adjusted OR	aOR 95% CI^a^
Catchment births per annum	**0.037**		
< 50		1.00	
50-100	**0.009**	46.81	[2.64-830]
100-150	**0.002**	89.06	[5.06-1569]
150-200	**0.024**	18.34	[1.47-229]
> 200	**0.004**	47.17	[3.32-670]
Time to nearest C-section facility	**0.003**		
< 0.5 h	0.128	4.70	[0.64-34.6]
0.5-1 h		1.00	
1-1.5 h	0.051	4.28	[1.00-18]
1.5-2 h	**0.035**	15.18	[1.21-189]
> 2 h	**0.000**	80.26	[6.79-948]
SES (IRSD deciles)	0.627		
6, 7 Less disadvantaged			1.00
5	0.206	32.91	[0.15-7434]
4	0.205	29.47	[0.16-5502]
3	0.288	17.52	[0.08-346]
1, 2 Most disadvantaged	0.360	11.32	[0.06-2043]
Jurisdiction categories	**0.001**		
VIC WAplus		1.00	
NSW QLD SA	**0.001**	11.34	[2.88-44.7]

^a^aOR 95% CI = Adjusted Odds Ratio 95% Confidence Interval

Bold indicates significant effects or significant aOR (*p* < 0.05)

## Discussion

This paper has described the association between birthing services in rural and remote Australia and characteristics of the catchment populations representing need: birth numbers, vulnerability and isolation so as to identify possible disparities in maternity service distribution.

The Australian National Maternity Services Plan acknowledged the considerable health inequalities and social disadvantage of Aboriginal and Torres Strait Islander people and rural and remote communities which is compounded by the limited provision of quality maternity care and the restricted birthing choices [20]. The purpose of this paper is to describe the association between birthing services in rural and remote Australia and the characteristics of the catchment populations in relation to equity of access.

This study defined catchments using one-hour travel time for Australian health facilities in rural and remote areas and then ascertained their level of maternity service provision. There is some evidence to suggest that distance is associated with maternal and neonatal outcomes, with Canadian studies indicating that women who have to travel more than one-hour to access birthing services have worse outcomes [33–35]. This study found that the number of births in a catchment was the strongest predictor for distinguishing between facilities that offered birthing and those that did not, and between those that offered C-section and those that only offered birthing. Our study demonstrated that increasing numbers of births significantly increased the likelihood of a higher level of service. However, as illustrated in Figs. 1 and 2 (and Additional file 5: Figure S1) there was an overlap in numbers of births in birthing and non-birthing facilities, and those with and without C-section.

In Australia there are well known poorer outcomes for those who are financially disadvantaged, who live in the non-metropolitan areas and who are from minority groups. Of the factors explored in this paper, only increasing birth numbers were consistently associated with higher levels of service. Other aspects of population-based need were not consistently associated with the distribution of services. For example, our proxy measure for isolation, the time it takes to travel by road to the nearest C-section facility revealed an unexpectedly mixed picture. Very remote communities are less likely to have any type of birthing service compared to less remote communities. Rural health facilities 1 to 2 h away from a C-section service are more likely to have birthing compared to those closer to C-section facilities. This means that being one hour or less from a C-section facility (i.e., within its catchment area) lessens your likelihood of being a C-section facility. This suggests that, controlling for other factors, once a birthing facility in rural and remote Australia is more than an hour from a C-section facility, health planners are less likely to retain birthing facilities that are not C-section capable. This is despite data showing that birthing without a C-section service produces better outcomes than no services at all [33].

Every health facility in our study was in the lowest 70% of the standardised SES index (the IRSD) in Australia, and more than 73% were in the lowest 40%. However, SES as a measure of vulnerability did not predict the level of service, after controlling for other catchment characteristics. Australian and international studies have demonstrated that maternal SES is a powerful determinant of adverse health outcomes for mothers [36] and newborns [37–39]. Similarly, Indigeneity is a significant determinant of perinatal morbidity in Australia [37]. However, the proportion of Aboriginal and Torres Strait Islander people in a catchment, again a measure of vulnerability, was not associated with the level of birthing service, suggesting that the current provision of birthing services in Australia does not accommodate the increased vulnerability and risk associated with higher proportions of Aboriginal and Torres Strait Islanders.

The provision of health services in Australia is undertaken by its eight jurisdictions. These data show that for populations living in rural or remote areas, the jurisdiction of residence has a strong influence on the level of maternity service provided. Geography, population distribution, government policy and service delineations contribute to this phenomenon. The Australian National Maternity Services Plan [14, 20] attempted to address this problem in providing an evidence-base to assist with more consistent decisions.

### Limitations

There are a number of limitations to this study. The sustainability of health care facilities is dependent on many factors in rural and remote areas. The existing maternity service levels were identified by our team in 2010 and any limitations associated with this are discussed in Longman et al. [31, 32]. Since that time, some birthing facilities have closed, opened or changed their service level either permanently or temporarily. Several of these facilities have been identified as ‘discordant sites’ in the regression analyses, perhaps reflecting their changes in service levels since 2010.

Interpretation of the results of Stage 1 models (birthing/no birthing) is complicated by the definition of catchment. The decision to give priority to the catchments for facilities that currently undertook birthing over non-birthing facilities was to increase the precision of the catchments. However, a number of non-birthing services were excluded that may have played a role in birthing service provision.

## Conclusion

The results of these analyses indicate that the equitable planning and maintenance of rural and remote Australian maternity services assessed at a population level is sub-optimal. This finding is supported by numerous reports and research demonstrating that birthing outcomes consistently show that rural and remote Australians have worse outcomes compared to urban families [40, 41]. The Australian National Maternity Services Plan highlighted the potential benefits of rural maternity service planning tools for rural communities to “*develop a rigorous methodology to assist in future planning for maternity care, including in rural and remote communities*” [20]. This research reinforces the justification of such a methodology. This study found that the provision of maternity services in rural and remote Australia is not based solely on the numbers of births, and provides minimal adjustment for needs of vulnerable and isolated rural and remote populations. In addition, services are influenced by jurisdictions.

## Additional files

Additional file 1: Table S1. Descriptive statistics and the tests of association for Stage 1 Modelling - birthing facilities versus non-birthing facilities. (DOCX 33 kb)
Additional file 2: Table S2. Results of the univariable logistic models for Stage 1 Modelling - birthing facilities versus non-birthing facilities. (DOCX 37 kb)
Additional file 3: Table S3. Descriptive characteristics of the facilities as well as results of tests of association and trend for Stage 2: facilities offering birthing with or without C-section. (DOCX 35 kb)
Additional file 4: Table S4. Summarised results of univariable logistic regression for Stage 2: facilities offering birthing with or without C-section. (DOCX 36 kb)
Additional file 5: Figure S1. Annual birth numbers (5 year average) of catchments for no birthing, no C-Section and C-Section birthing facilities. (DOCX 41 kb)

## Abbreviations

95% CI: 95% Confidence Interval
ABS: Australian Bureau of Statistics
aOR: Adjusted Odds ratio
AUC: Area under the Curve
CCD: Census Collection Districts
chisq: Chi square statistic
C-section: Caesarean section
df: Degrees of freedom
ERP: Estimated Resident Population
GIS: Geographic information systems
h: hours
IRSD: Socio-economic indexes for areas index of relative socio economic disadvantage
Km: Kilometres
LRT: Likelihood Ratio Test
MB: Mesh block
NSW: New South Wales
NT: Northern Territory
OR: Odds ratio
Qld: Queensland
RA: Australian Bureau of Statistics Remoteness Area Structure
RBI: Rural birthing index
Ref: Reference category
R_Nag_^2^: Nagelkerke pseudo R^2^
SA: South Australia
SD: Standard deviation
SES: Socioeconomic status
SLA: Statistical Local Areas
TAS: Tasmania
URP: Usual Resident Population
Vic: Victoria
WA: Western Australia
WAPlus: WA, TAS, NT combined
