# Supplementary material for: The distribution of maternity services across rural and remote Australia: does it reflect population need?
Source: BMC Health Serv Res. 2017 Feb 23;17:163. doi: 10.1186/s12913-017-2084-8 (PMC5324256; doi:10.1186/s12913-017-2084-8)
Supplement: Additional file 1: Table S1. — Descriptive statistics and the tests of association for Stage 1 Modelling - birthing facilities versus non-birthing facilities. (DOCX 33 kb) [file 12913_2017_2084_MOESM1_ESM.docx]

# **Additional file 1 Table S1** Descriptive statistics and the tests of association for Stage 1 Modelling - birthing facilities versus non-birthing facilities.

| Categories | Non-birthing | | Birthing | | Total |  |  |  |
| --- | --- | --- | --- | --- | --- | --- | --- | --- |
|  | n | Row % | n | Row % | n | Col % | Chisq | P value |
|  |  |  |  |  |  |  |  |  |
| Births / year |  |  |  |  |  |  |  |  |
| <=50 | 116 | 91% | 11 | 9% | 127 | 49% | 132.4 | <0.001 |
| 50-99 | 23 | 50% | 23 | 50% | 46 | 18% |  |  |
| 100-149 | 8 | 27% | 22 | 73% | 30 | 12% |  |  |
| 150-199 | 4 | 16% | 21 | 84% | 25 | 10% |  |  |
| 200-249 | 0 | 0% | 20 | 100% | 20 | 8% |  |  |
| 250-299 | 0 | 0% | 8 | 100% | 8 | 3% |  |  |
| > 300 | 0 | 0% | 3 | 100% | 3 | 1% |  |  |
|  |  |  |  |  |  |  |  |  |
| SES (IRSD) |  |  |  |  |  |  |  |  |
| 6-7 Least disadvantaged | 21 | 84% | 4 | 16% | 25 | 10% | 24.8 | <0.001 |
| 5 | 29 | 66% | 15 | 34% | 44 | 17% |  |  |
| 4 | 32 | 54% | 40 | 46% | 72 | 28% |  |  |
| 3 | 33 | 54% | 28 | 46% | 61 | 24% |  |  |
| 2 | 13 | 43% | 17 | 57% | 30 | 12% |  |  |
| 1 most disadvantaged | 23 | 85% | 4 | 15% | 27 | 10% |  |  |
|  |  |  |  |  |  |  |  |  |
| Jurisdiction |  |  |  |  |  |  |  |  |
| NSW | 41 | 59% | 28 | 41% | 69 | 27% | 17.8 | <0.001 |
| QLD | 30 | 63% | 18 | 38% | 48 | 19% |  |  |
| VIC | 16 | 39% | 25 | 61% | 41 | 16% |  |  |
| SA | 16 | 44% | 20 | 56% | 36 | 14% |  |  |
| WA | 30 | 68% | 14 | 31% | 44 | 17% |  |  |
| NT | 10 | 83% | 2 | 17% | 12 | 5% |  |  |
| TAS | 8 | 89% | 1 | 11% | 9 | 4% |  |  |
|  |  |  |  |  |  |  |  |  |
| Time to C-section (hours) |  |  |  |  |  |  |  |  |
| Up to 1 hr | 7 | 13% | 47 | 87% | 54 | 21% | 62.4 | <0.001 |
| 1-2 | 83 | 68% | 39 | 32% | 122 | 47% |  |  |
| 2-3 | 27 | 84% | 5 | 16% | 32 | 12% |  |  |
| 3-4 | 8 | 53% | 7 | 47% | 15 | 6% |  |  |
| 4 and more | 26 | 72% | 10 | 28% | 36 | 14% |  |  |
|  |  |  |  |  |  |  |  |  |
| Remoteness |  |  |  |  |  |  |  |  |
| RA 2 Inner regional | 14 | 28% | 36 | 33% | 50 | 19% | 33.6 | <0.001 |
| RA 3 Outer regional | 73 | 57% | 55 | 43% | 128 | 49% |  |  |
| RA 4 Remote | 39 | 76% | 12 | 24% | 51 | 20% |  |  |
| RA 5 Very remote | 25 | 83% | 5 | 17% | 30 | 12% |  |  |
|  |  |  |  |  |  |  |  |  |
| Aboriginal & Torres Strait Islander |  |  |  |  |  |  |  |  |
| < 2.5% | 62 | 59% | 44 | 41% | 106 | 41% | 9.9 | 0.041 |
| 2.5%-5% | 27 | 47% | 31 | 53% | 58 | 22% |  |  |
| 5%-10% | 31 | 65% | 17 | 35% | 48 | 18% |  |  |
| 10%-25% | 11 | 50% | 11 | 50% | 22 | 9% |  |  |
| >= 25% | 20 | 80% | 5 | 20% | 25 | 10% |  |  |

# SES levels 8-10 [least disadvantaged) not present in facilities modelled
