# Supplementary material for: The distribution of maternity services across rural and remote Australia: does it reflect population need?
Source: BMC Health Serv Res. 2017 Feb 23;17:163. doi: 10.1186/s12913-017-2084-8 (PMC5324256; doi:10.1186/s12913-017-2084-8)
Supplement: Additional file 2: Table S2. — Results of the univariable logistic models for Stage 1 Modelling - birthing facilities versus non-birthing facilities. (DOCX 37 kb) [file 12913_2017_2084_MOESM2_ESM.docx]

**Additional file 2. Table S2** Results of the univariable logistic models for Stage 1 Modelling - birthing facilities versus non-birthing facilities

| Model components LRT | Chisq | Wald | P | OR | 95% CI | % Agree | R_Neg_^2^ | AUC | Discordant sites |
| --- | --- | --- | --- | --- | --- | --- | --- | --- | --- |
|  |  |  |  |  |  |  |  |  |  |
| Births |  |  |  |  |  |  |  |  |  |
| Continuous (÷10) | 152.8 | 67.4 | 0.000 | 1.37 | 1.27-1.47 | 81.5 | 0.60 | 0.91 | 48 |
| 4 categories | 149.7 | 84.0 | 0.000 |  |  | 82.2 | 0.59 | 0.89 | 46 |
| <50 |  |  |  | 1.00 |  |  |  |  |  |
| 50-100 |  | 29.8 | 0.000 | 10.55 | 4.52-24.58 |  |  |  |  |
| 100-150 |  | 42.0 | 0.000 | 29.00 | 10.47-80.29 |  |  |  |  |
| >150 |  | 65.7 | 0.000 | 137.09 | 41.70-450.72 |  |  |  |  |
|  |  |  |  |  |  |  |  |  |  |
| Females 14-44 |  |  |  |  |  |  |  |  |  |
| Continuous % | 70.1 | 42.8 | 0.000 | 0.74 | 0.67-0.81 | 69.1 | 0.32 | 0.79 | 80 |
|  |  |  |  |  |  |  |  |  |  |
| Travel time |  |  |  |  |  |  |  |  |  |
| 1 hour categories | 66.3 | 44.9 | 0.000 |  |  | 73.7 | 0.30 | 0.74 | 68 |
| < 1 hour |  | 35.0 | 0.000 | 14.29 | 5.92-34.47 |  |  |  |  |
| 1-2hrs |  |  |  | 1.00 |  |  |  |  |  |
| 2-3 hrs |  | 3.2 | 0.076 | 0.39 | 0.14-1.10 |  |  |  |  |
| 3-4 hrs |  | 1.3 | 0.261 | 1.86 | 0.63-5.50 |  |  |  |  |
| > 4 hours |  | 0.2 | 0.633 | 0.82 | 0.36-1.86 |  |  |  |  |
|  |  |  |  |  |  |  |  |  |  |
| SES |  |  |  |  |  |  |  |  |  |
| 4 categories | 25.1 | 20.6 | 0.000 |  |  | 58.3 | 0.12 | 0.65 | 108 |
| 6-7 highest |  |  |  | 1.00 |  |  |  |  |  |
| 3-5 mid |  | 2.5 | 0.114 | 2.72 | 0.79-9.36 |  |  |  |  |
| 2 low-mid |  | 9.4 | 0.002 | 5.72 | 1.88-17.40 |  |  |  |  |
| 1 lowest |  | 0.0 | 0.906 | 0.91 | 0.20-4.12 |  |  |  |  |
|  |  |  |  |  |  |  |  |  |  |
| Jurisdiction |  |  |  |  |  |  |  |  |  |
| 7 categories | 18.7 | 16.2 | 0.005 |  |  | 61.8 | 0.09 | 0.64 | 99 |
| NSW (Ref) |  |  |  | 1.00 |  |  |  |  |  |
| QLD |  | 0.1 | 0.737 | 0.88 | 0.41-1.87 |  |  |  |  |
| Vic |  | 4.2 | 0.040 | 2.29 | 1.04-5.04 |  |  |  |  |
| SA |  | 2.1 | 0.146 | 1.83 | 0.81-4.13 |  |  |  |  |
| WA |  | 0.9 | 0.348 | 0.68 | 0.31-1.51 |  |  |  |  |
| NT |  | 2.3 | 0.131 | 0.29 | 0.06-1.44 |  |  |  |  |
| Tas |  | 2.4 | 0.119 | 0.18 | 0.02-1.55 |  |  |  |  |
|  |  |  |  |  |  |  |  |  |  |
| Remoteness |  |  |  |  |  |  |  |  |  |
| 4 categories | 35.0 | 29.8 | 0.000 |  |  | 66.8 | 0.17 | 0.69 | 86 |
| RA 2 Inner regional |  |  |  | 1.00 |  |  |  |  |  |
| RA 3 outer regional |  | 11.5 | 0.001 | 0.29 | 0.14-0.60 |  |  |  |  |
| RA 4 remote |  | 21.7 | 0.000 | 0.12 | 0.05-0.29 |  |  |  |  |
| RA5 Very remote |  | 19.2 | 0.000 | 0.08 | 0.02-0.24 |  |  |  |  |
|  |  |  |  |  |  |  |  |  |  |
| Aboriginal &Torres Strait Islander |  |  |  |  |  |  |  |  |  |
| Continuous % | 8.6 | 6.1 | 0.003 | 0.98 | 0.96-1.00 | 58.5 | 0.04 | 0.56 | 108 |
| 5 categories (%) | 10.0 | 9.0 | 0.041 |  |  | 58.3 | 0.05 | 0.60 | 108 |
| 0-2.49 |  | 2.1 | 0.143 | 0.62 | 0.32-1.18 |  |  |  |  |
| 2.5-4.99 (Ref) |  |  |  | 1.00 |  |  |  |  |  |
| 5-9.99 |  | 3.4 | 0.065 | 0.48 | 0.22-1.05 |  |  |  |  |
| 10-24.99 |  | 0.1 | 0.783 | 0.87 | 0.33-2.33 |  |  |  |  |
| >=25 |  | 7.3 | 0.007 | 0.22 | 0.07-0.66 |  |  |  |  |
