# Supplementary material for: The distribution of maternity services across rural and remote Australia: does it reflect population need?
Source: BMC Health Serv Res. 2017 Feb 23;17:163. doi: 10.1186/s12913-017-2084-8 (PMC5324256; doi:10.1186/s12913-017-2084-8)
Supplement: Additional file 3: Table S3. — Descriptive characteristics of the facilities as well as results of tests of association and trend for Stage 2: facilities offering birthing with or without C-section. (DOCX 35 kb) [file 12913_2017_2084_MOESM3_ESM.docx]

**Additional file 3. Table S3** Descriptive characteristics of the facilities as well as results of tests of association and trend for Stage 2: facilities offering birthing with or without C-section

|  | No C-Section | | | | | C-section | | | | | | Total | | | | | | Chisq  P value | | | Linear Trend  P value | | |  |
| --- | --- | --- | --- | --- | --- | --- | --- | --- | --- | --- | --- | --- | --- | --- | --- | --- | --- | --- | --- | --- | --- | --- | --- | --- |
|  | n | | Row% | | | n | | | Row % | | | n | | | Col % | | |  |  |  |  |  |  |  |
|  |  | | |  | | |  | | |  | | |  | | |  | | |  | | |  | |  |
| Birth numbers |  | | |  | | |  | | |  | | |  | | |  | | |  | | |  | |  |
| <=50 | 9 | | | 81.8% | | | 2 | | | 18.2% | | | 11 | | | 10.2% | | | 0.003 | | | 0.027 | |  |
| 50-100 | 8 | | | 34.8% | | | 15 | | | 65.2% | | | 23 | | | 21.3% | | |  | | |  | |  |
| 100-150 | 4 | | | 18.2% | | | 18 | | | 81.8% | | | 22 | | | 20.4% | | |  | | |  | |  |
| 150-200 | 7 | | | 33.3% | | | 14 | | | 66.7% | | | 21 | | | 19.4% | | |  | | |  | |  |
| >200 | 7 | | | 22.6% | | | 24 | | | 77.4% | | | 31 | | | 28.7% | | |  | | |  | |  |
|  |  | | |  | | |  | | |  | | |  | | |  | | |  | | |  | |  |
| SES (IRSD) |  | | |  | | |  | | |  | | |  | | |  | | |  | | |  | |  |
| 6-7 Least disadvantaged | 3 | | | 75.0% | | | 1 | | | 25.0% | | | 4 | | | 3.7% | | | 0.366 | | | 0.235 | |  |
| 4-5 | 4 | | | 26.7% | | | 11 | | | 73.3% | | | 15 | | | 13.9% | | |  | | |  | |  |
| 3 | 13 | | | 32.5% | | | 27 | | | 67.5% | | | 40 | | | 37.0% | | |  | | |  | |  |
| 2 | 10 | | | 35.7% | | | 18 | | | 64.3% | | | 28 | | | 25.9% | | |  | | |  | |  |
| 1 most disadvantaged | 5 | | | 23.8% | | | 16 | | | 76.2% | | | 21 | | | 19.4% | | |  | | |  | |  |
|  |  | | |  | | |  | | |  | | |  | | |  | | |  | | |  | |  |
| Time to C-section |  | | |  | | |  | | |  | | |  | | |  | | |  | | |  | |  |
| up to half hour | 2 | | | 18.2% | | | 9 | | | 81.8% | | | 11 | | | 10.2% | | | 0.001 | | | 0.002 | |  |
| 0.5-1 | 20 | | | 55.6% | | | 16 | | | 44.4% | | | 36 | | | 33.3% | | |  | | |  | |  |
| 1-1.5 | 10 | | | 37.0% | | | 17 | | | 63.0% | | | 27 | | | 25.0% | | |  | | |  | |  |
| 1.5 and more | 3 | | | 8.8% | | | 31 | | | 91.2% | | | 34 | | | 31.5% | | |  | | |  | |  |
|  |  | | |  | | |  | | |  | | |  | | |  | | |  | | |  | |  |
| Jurisdiction |  | | |  | | |  | | |  | | |  | | |  | | |  | | |  | |  |
| NSW | 8 | | | 28.6% | | | 20 | | | 71.4% | | | 28 | | | 25.9% | | | 0.005 | | | 0.310 | |  |
| QLD | 2 | | | 11.1% | | | 16 | | | 88.9% | | | 18 | | | 16.7% | | |  | | |  | |  |
| VIC | 14 | | | 56.0% | | | 11 | | | 44.0% | | | 25 | | | 23.1% | | |  | | |  | |  |
| SA | 3 | | | 15.0% | | | 17 | | | 85.0% | | | 20 | | | 18.5% | | |  | | |  | |  |
| WAplus | 8 | | | 47.1% | | | 9 | | | 52.9% | | | 17 | | | 15.7% | | |  | | |  | |  |
| WA | 7 | | | 50.0% | | | 7 | | | 50.0% | | | 14 | | | 13.0% | | |  | | |  | |  |
| NT | 0 | | | 0.0% | | | 2 | | | 1.0% | | | 2 | | | 1.9% | | |  | | |  | |  |
| TAS | 1 | | | 1.0% | | | 0 | | | 0.0% | | | 1 | | | 0.9% | | |  | | |  | |  |
| Jurisdiction (combine) |  | | |  | | |  | | |  | | |  | | |  | | |  | | |  | |  |
| Vic WA plus | 22 | | | 52.4% | | | 20 | | | 47.6% | | | 42 | | | 38.9% | | | 0.000 | | | 0.000 | |  |
| NSW SA QLD | 13 | | | 19.7% | | | 53 | | | 80.3% | | | 73 | | | 61.1% | | |  | | |  | |  |
|  |  | | |  | | |  | | |  | | |  | | |  | | |  | | |  | |  |
| Aboriginal & Torres Strait Islander | |  | | |  | | |  | | |  | | |  | | |  | | |  | | |  | |
| <2.5% | 19 | | | 43.2% | | | 25 | | | 56.8% | | | 44 | | | 40.7% | | | 0.100 | | | 0.014 | |  |
| 2.5-5% | 9 | | | 29.0% | | | 22 | | | 71.0% | | | 31 | | | 28.7% | | |  | | |  | |  |
| 5-10% | 6 | | | 35.3% | | | 11 | | | 64.7% | | | 17 | | | 15.7% | | |  | | |  | |  |
| 10-25% | 1 | | | 9.1% | | | 10 | | | 90.9% | | | 11 | | | 10.2% | | |  | | |  | |  |
| >25% | 0 | | | 0% | | | 5 | | | 100% | | | 5 | | | 4.6% | | |  | | |  | |  |
|  |  | | |  | | |  | | |  | | |  | | |  | | |  | | |  | |  |
| Remoteness |  | | |  | | |  | | |  | | |  | | |  | | |  | | |  | |  |
| RA 2 Inner regional | 15 | | | 41.7% | | | 21 | | | 58.3% | | | 36 | | | 33.3% | | | 0.157 | | | 0.035 | |  |
| RA 3 Outer regional | 18 | | | 32.7% | | | 37 | | | 67.3% | | | 55 | | | 50.9% | | |  | | |  | |  |
| RA 4 Remote | 2 | | | 16.7% | | | 10 | | | 83.3% | | | 12 | | | 11.1% | | |  | | |  | |  |
| RA 5 Very remote | 0 | | | 0% | | | 5 | | | 100% | | | 5 | | | 4.6% | | |  | | |  | |  |
| Remoteness (2 cat) |  | | |  | | |  | | |  | | |  | | |  | | |  | | |  | |  |
| RA 2-3 Rural | 33 | | | 36.3% | | | 58 | | | 63.7% | | | 91 | | | 84.3% | | | 0.053 | | | 0.048 | |  |
| RA 4-5 Remote | 2 | | | 11.8% | | | 15 | | | 88.2% | | | 17 | | | 15.7% | | |  | | |  | |  |
