# Supplementary material for: The distribution of maternity services across rural and remote Australia: does it reflect population need?
Source: BMC Health Serv Res. 2017 Feb 23;17:163. doi: 10.1186/s12913-017-2084-8 (PMC5324256; doi:10.1186/s12913-017-2084-8)
Supplement: Additional file 4: Table S4. — Summarised results of univariable logistic regression for Stage 2: facilities offering birthing with or without C-section. (DOCX 36 kb) [file 12913_2017_2084_MOESM4_ESM.docx]

**Additional file 4. Table S4** Summarised results of univariable logistic regression for Stage 2: facilities offering birthing with or without C-section

| Model components | LRT Chisq | Wald | P | OR | 95% CI | % agree | R_Nag_^2^ | AUC | Discordant sites |
| --- | --- | --- | --- | --- | --- | --- | --- | --- | --- |
|  |  |  |  |  |  |  |  |  |  |
| Birth numbers | 6.59 | 6.03 | 0.014 | 1.08 | 1.01- 1.14 | 67.6 | 0.083 | 0.64 | 35 |
| Birth numbers 4 category | 14.47 | 10.86 | 0.013 |  |  | 74.1 | 0.175 | 0.67 | 28 |
| ≤ 50 |  |  |  | 1.00 |  |  |  |  |  |
| 51-100 |  | 5.67 | 0.017 | 8.44 | 1.46-48.85 |  |  |  |  |
| 101-150 |  | 9.87 | 0.002 | 20.25 | 3.10-132.25 |  |  |  |  |
| >150 |  | 8.84 | 0.003 | 12.21 | 2.35-63.61 |  |  |  |  |
| Birth numbers 5 category | 15.19 | 11.51 | 0.021 |  |  | 74.1 | 0.183 | 0.83 | 28 |
| ≤ 50 |  |  |  | 1.00 |  |  |  |  |  |
| 51-100 |  | 5.67 | 0.017 | 8.44 | 1.46-48.85 |  |  |  |  |
| 101-150 |  | 9.87 | 0.002 | 20.25 | 3.10-132.25 |  |  |  |  |
| 150-200 |  | 5.85 | 0.016 | 9.00 | 1.52-53.40 |  |  |  |  |
| >200 |  | 9.41 | 0.002 | 15.43 | 2.69-88.63 |  |  |  |  |
|  |  |  |  |  |  |  |  |  |  |
| SES Continuous | 1.65 | 1.61 | 0.205 | 1.26 | 0.88- 1.80 | 67.6 | 0.021 | 0.55 | 35 |
| SES 5 category | 4.16 | 3.58 | 0.465 |  |  | 69.4 | 0.053 | 0.59 | 33 |
|  |  |  |  |  |  |  |  |  |  |
| Time |  |  |  |  |  |  |  |  |  |
| Time (minutes) | 9.61 | 5.59 | 0.018 | 1.01 | 1.00- 1.02 | 67.6 | 0.119 | 0.65 | 35 |
| Time (hours) 4 categories | 20.28 | 15.51 | 0.001 |  |  | 67.6 | 0.239 | 0.75 | 35 |
| up to 0.5hr |  | 4.12 | 0.042 | 5.63 | 1.06-29.80 |  |  |  |  |
| 0.5-1 |  |  |  | 1.00 |  |  |  |  |  |
| 1-1.5 |  | 2.09 | 0.148 | 2.13 | 0.77- 5.90 |  |  |  |  |
| 1.5 and more |  | 13.69 | 0.000 | 12.92 | 3.33-50.08 |  |  |  |  |
| Time([hours) 5 category | 21.62 | 14.53 | 0.006 |  |  | 67.6 | 0.253 | 0.75 | 35 |
| up to 0.5hr |  | 4.12 | 0.042 | 5.63 | 1.06-29.80 |  |  |  |  |
| 0.5-1 |  |  |  | 1.00 |  |  |  |  |  |
| 1-1.5 |  | 2.09 | 0.148 | 2.13 | 0.77- 5.90 |  |  |  |  |
| 1.5-2 |  | 4.71 | 0.030 | 6.25 | 1.20-32.69 |  |  |  |  |
| 2 & more |  | 9.20 | 0.002 | 26.25 | 3.18-216.74 |  |  |  |  |
|  |  |  |  |  |  |  |  |  |  |
| RA (Rural, Remote) | 4.55 | 32.43 | 0.064 | 4.27 | 0.92-19.83 | 67.6 | 0.058 | 0.57 | 35 |
|  |  |  |  |  |  |  |  |  |  |
| Aboriginal and Torres Strait Islander % linear | 9.89 | 5.09 | 0.024 | 1.16 | 1.02- 1.31 | 67.6 | 0.122 | 0.65 | 35 |
| % 4 category | 8.98 | 5.86 | 0.119 |  |  | 67.6 | 0.111 | 0.64 | 35 |
| <2.5% |  |  |  | 1.00 |  |  |  |  |  |
| 2.5-5 |  | 1.54 | 0.215 | 1.86 | 0.70- 4.94 |  |  |  |  |
| 5-10 |  | 0.20 | 0.575 | 1.39 | 0.44- 4.44 |  |  |  |  |
| 10+ |  | 2.69 | 0.024 | 11.40 | 1.38-94.06 |  |  |  |  |
|  |  |  |  |  |  |  |  |  |  |
| Jurisdiction (5) | 15.29 | 13.09 | 0.011 |  |  | 67.6 | 0.184 | 0.72 | 35 |
| NSW |  |  |  | 1.00 |  |  |  |  |  |
| QLD |  | 1.83 | 0.176 | 3.20 | 0.59-17.22 |  |  |  |  |
| VIC |  | 3.97 | 0.046 | 0.31 | 0.10- 0.98 |  |  |  |  |
| SA |  | 1.18 | 0.277 | 2.27 | 0.52- 9.92 |  |  |  |  |
| WAplus |  | 1.55 | 0.213 | 0.45 | 0.13- 1.58 |  |  |  |  |
|  |  |  |  |  |  |  |  |  |  |
| Percent females 15-44 | 0.59 | 0.59 | 0.444 | 0.94 | 0.81- 1.10 | 67.6 | 0.008 | 0.54 | 35 |
