# Supplementary material for: The distribution of maternity services across rural and remote Australia: does it reflect population need?
Source: BMC Health Serv Res. 2017 Feb 23;17:163. doi: 10.1186/s12913-017-2084-8 (PMC5324256; doi:10.1186/s12913-017-2084-8)
Supplement: Additional file 5: Figure S1. — Annual birth numbers (5 year average) of catchments for no birthing, no C-Section and C-Section birthing facilities. (DOCX 41 kb) [file 12913_2017_2084_MOESM5_ESM.docx]

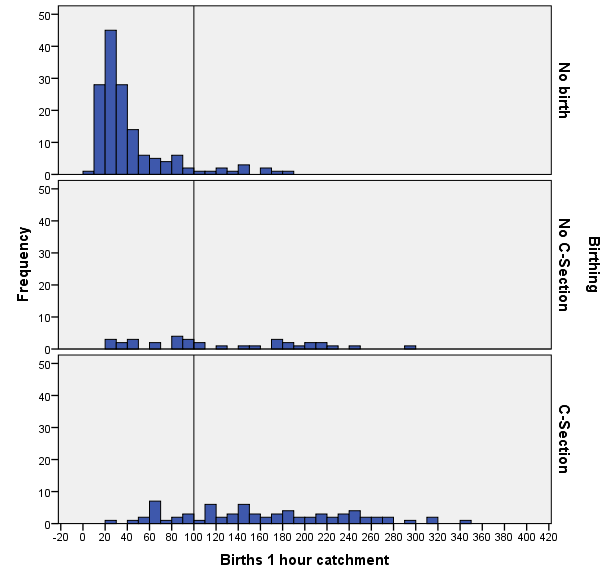


Additional File 5. Fig S1 Annual birth numbers (5 year average) of catchments for no birthing, no C-Section and C-Section birthing facilities.
